# Properties of Adhesive Mortars Using Waste Glass

**DOI:** 10.3390/ma17153853

**Published:** 2024-08-03

**Authors:** Galyna Kotsay, Wiktor Szewczenko

**Affiliations:** Faculty of Civil Engineering, Mechanics and Petrochemistry, Warsaw University of Technology, Łukasiewicza St. 17, 09-400 Płock, Poland; galyna.kotsay@pw.edu.pl

**Keywords:** waste glass, water glass, adhesive mortar, alkaline activity, polyalkaline effect, tensile strength

## Abstract

This study investigates the use of waste glass as an active aggregate in glass polymers based on water glass, aiming to enhance the sustainability of construction materials by utilizing recyclable waste. Methodologically, the research employs a combination of water glass as a binder with waste glass, analyzing their chemical interaction and the resulting mechanical properties. The primary findings reveal that the inclusion of finely ground waste glass not only promotes the polycondensation and hardening processes of water glass but also significantly influences the adhesive and cohesive strengths of the developed glass polymers. After 7 days of hardening, the tensile strength of these materials exceeds that of standard concrete with values reaching up to 4.11 MPa, indicating strong adhesion capabilities that could pull out fragments of the concrete substrate. Conclusively, the study underscores the potential of waste glass in improving the structural and economic efficiencies of building materials, contributing to a reduction in landfill waste and offering a promising avenue for the innovative use of recyclable materials in construction.

## 1. Introduction

Waste glass is an ideal recyclable secondary raw material [1,2,3]. It boasts durability and chemical resistance, posing no threat to the environment, yet it burdens landfills as it is not biodegradable [4,5,6,7]. As glass production increases annually, so does the volume of cullet [8]. For this reason, the efficient management of glass waste has become a key challenge in waste management [9,10]. The management strategy depends on the type of glass [11]. However, particular difficulties are associated with the unstable chemical composition of the cullet and the challenge of segregating different types of glass during disposal [5,6]. Currently, most recyclable glass is container glass, which is relatively easy to process due to its uniform composition. Using waste glass in construction can significantly contribute to reducing landfill waste and lowering material costs as well as enhance the sustainability of the construction industry by creating innovative materials.

One effective method for using cullet is to produce glass fiber, rock wool, abrasives, and road paints [12,13]. Many publications have proposed using waste glass as an aggregate or a non-clinker component in cement [14,15,16,17,18,19,20,21,22,23,24,25,26,27,28,29]. However, the use of glass in cement products is limited due to its high alkali content, which in turn can cause an alkali–silica reaction (ASR) and reduce the strength of the resulting products [30,31,32]. There is growing interest in using waste glass in alkali-activated materials or geopolymers, where the acceptable glass content ranges from 10 to 50%. It is currently relevant to develop new building materials using waste glass combined with water glass [33,34,35,36,37].

Water glass is a binder with high strength properties and considerable acid resistance except against hydrofluoric acid [36,38]. In construction, sodium and potassium water glass are used for producing fireproof materials, adhesive mortars, wood impregnation, concrete surface hardening, the activation of mineral additives, and a component in geopolymers [39,40,41,42,43,44]. Its application depends on the modulus (the molar ratio of silicate to sodium or potassium oxide), concentration, and density. In construction, water glass is most commonly used with a modulus ranging from 1.5 to 3.5 and a density from 1.3 to 1.4 kg/dm^3^ [36,45,46]. One of the most important properties of this material is its adhesive ability, characterized by adhesive strength (fad), and as water glass hardens and acquires the properties of a solid body, its structural strength, or cohesive strength (fcoh), also becomes crucial [38].

The setting and hardening mechanisms of water glass differ significantly from those of ordinary cement. While the setting process of cement is based on hydration reactions, the setting process of water glass relies on polycondensation reactions. Polycondensation leads to the formation of amorphous or semi-crystalline silicon tetrahedral groups, which are linked by alternating common oxygen atoms. Given its relatively high chemical activity, the interaction of water glass with various chemical reagents offers the possibility of obtaining materials with varying degrees of polymerization. The introduction of finely ground waste glass into water glass initiates a chemical interaction with the alkaline components of the glass powder, resulting in the formation of a new material, glass polymer (GP). The technological properties of this glass polymer depend heavily on the binding agent, water glass, and factors such as the type and surface area of the waste glass.

It is known that adding a filler to water glass promotes the processes of polycondensation and hardening of the water glass [36,47]. However, the potential use of waste glass as an aggregate is limited due to its high alkali content and the low adhesion of glass as an aggregate to cement slurry. Therefore, the purpose of this study was to combine waste glass as an aggregate with water glass as a binder to produce adhesive grouts. This research focuses on the adhesive and cohesive properties, aiming to determine the optimal amount of waste glass in the grouts tested.

## 2. Materials and Methods

This paper used waste glass produced by REWA [48] and sodium and potassium water glasses produced by the Chemical Plant “Rudniki” S.A. [49]. Their characteristics are presented in Table 1. The density and the Blaine-specific surface waste glass were performed according to the methods described in the standards [50,51].

The adhesive strength of hardened mortars on substrates was determined according to the standard [52]. As a substrate for determining the adhesion strength, a concrete slab measuring 300 × 500 × 50 mm^3^ with concrete compressive strength f_c_ = 70.5 MPa and tensile strength f_t_ ~4.0 MPa was used. The work investigated the effect of the composition of the glass polymer by changing the water glass to waste glass ratio from 1/1 to 1/3 on its adhesive (f_ad_) and cohesive (f_coh_) strength. Glass polymer (GP) was synthesized by mixing glass powder with water glass for 2–3 min until a homogeneous dense mass is obtained. Then, the glass polymer is applied to the surface of a pre-cleaned concrete slab, and the thickness of the applied layer is ~3 mm. After 30 days of hardening, a metal plate measuring 10 × 50 × 50 mm^3^ with a hole for a metal rod that is used to attach it to the press was glued to the surface of the glass polymer using epoxy glue. After the epoxy glue has hardened, cuts are made along the side walls of the metal square with an emery disk, separating a glass polymer sample with an area of 50 cm^2^. A total of 8 samples are obtained on the surface of the concrete slab.

**Figure 1:**
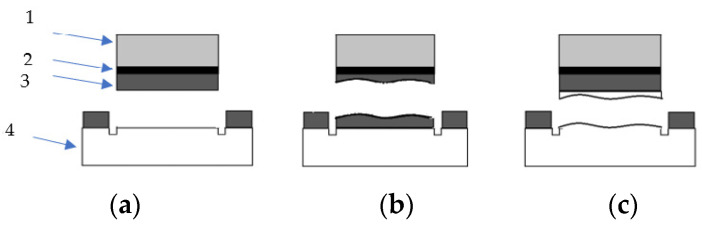
Fracture patterns (1—pull-head plate; 2—adhesive layer; 3—glass polymer; 4—concrete): (**a**) adhesion fracture—fracture at the interface between glass polymer and concrete; (**b**) cohesion fracture in glass polymer—fracture in the glass polymer itself; (**c**) cohesion fracture in concrete- fracture in the concrete itself.

Possible fracture patterns are given in Figure 1.

The Calmetrix I-Cal 2000 HPC calorimeter(Calmetrix Inc., located in Boston, Massachusetts, USA) was utilized to assess the impact of waste glass on the polycondensation reactions of water glass. Heat evolution was recorded for pastes with varying ratios of finely ground waste glass to water glass (1:1 and 1:2). The amount of heat released was monitored every 15 s over 100 h.

Alkaline activity was determined based on a series of six specimens according to [53]. For the analysis of alkali content, a flame photometer FP902 (PG Instruments Limited, Alma Park, Wibtoft, Leicestershire, UK) with an accuracy of ±0.5% was employed. Distilled water was used as an extractant to determine the alkaline activity of individual components and compositions of glass polymers. The results are presented in units of ppm/dm^2^ of paste.

Impact and compressive strength tests were measured for glass polymer specimens with dimensions of 10 mm × 10 mm × 80 mm. The strength tests were carried out 5, 9, and 16 days after forming. To research impact strength, a Charpy hammer was employed according to [54]. Compressive strength was determined using a ZD 10/90 static testing machine [55].

## 3. Results and Discussion

The research aimed to establish the role of the time factor and the glass polymer’s (GP) composition on its adhesion to the concrete surface. Table 2 shows the compositions and bond strengths studied. The use of the method described above for determining the adhesion strength of a glass polymer to a concrete surface (f_ad_) is based on determining the tensile strength of the GP/concrete composition. It is generally accepted that if the separation zone passes through the glass polymer, then the tensile strength will characterize the f_coh_ of the glass polymer. If the separation zone passes through concrete, then the tensile strength will characterize the cohesive strength of concrete (f_coh.c_).

Considering that tensile strength indirectly characterizes the material’s cohesive strength, i.e., its structural strength, it can be observed that over 28 days, the cohesive strength represented by f_coh_ for SWG and PWG increases significantly. Specifically, the data show that SWG alone and PWG alone see their tensile strengths increase to 0.65 MPa and 0.70 MPa, respectively, over a period of 28 days. This tenfold increase in the cohesive strength of water glass is associated with the polycondensation process of water glass. Moreover, the combined presence of SWG and PWG in different ratios increases cohesive strength over the first 7 days but shows a negligible change over the duration of 28 days when compared to using SWG or PWG individually.

**Figure 2:**
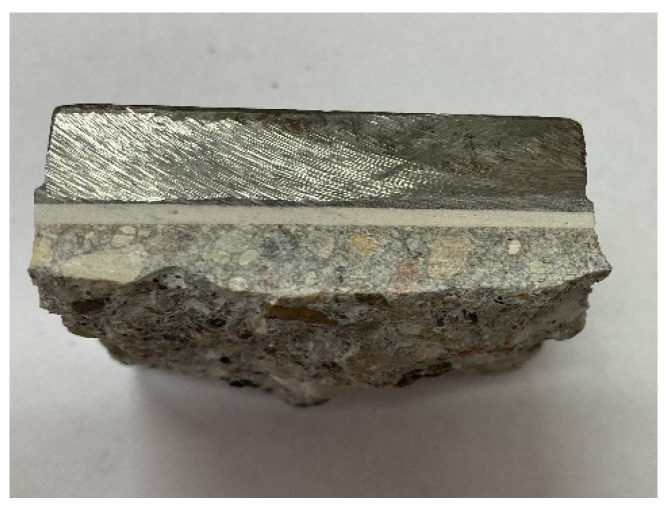
Sample of glass polymer glued to concrete surface after a tensile test.

The introduction of finely ground glass waste dramatically alters this trend. After 7 days of hardening, the tensile strength increases sharply, reaching values that exceed the tensile strength of standard concrete, which in our conditions is approximately 4 MPa. Specifically, a composite with 33.30% SWG and 67.70% glass waste reaches a tensile strength of about 4.11 MPa after 7 days, demonstrating a robust adhesion so powerful that the sample pulls out a fragment of the concrete substrate, as indicated in Figure 2.

A similar picture is observed for both SWG and PWG. Thus, introducing finely ground glass as an active filler into the composition of a glass polymer sharply increases its cohesive strength (f_coh_). In order to test this phenomenon, studies were carried out on the adhesive properties of a glass polymer using the example of tensile strength at bending. For this purpose, standard cement beams CEM I 32.5R measuring 40 × 40 × 160 mm^3^ were subjected to tensile bending testing. Then, both halves of the same sample were glued with a glass polymer of the appropriate composition and kept in air at room temperature for 28 days. After this, the glued beams were re-tested for tensile strength in bending, while the point of application of the force was located on the bonding line. The results showed that the failure plane was on the bonding line in all cases with glass polymer residues on both halves. This may indicate that the f_ad_ of the glass polymer in all cases exceeds it cohesive strength. At the same time, the compositions of glass polymers (SWG:GW/1:2) and (PWG:GW/1:2) are closest in value to the strength of the standard sample (f_t_ = 7.5 MPa), respectively, 6.5 and 5.1 MPa, as shown in Table 3. It should be emphasized that the use of dealcalized glass powder reduces the strength of glued samples by more than half, which may indicate the important role of alkalis in the structure of the glass polymer. In addition, it should be noted that potassium water glass is less effective than sodium glass.

As is known, alkalis in the composition of inorganic glasses significantly affect its properties [56,57]. Considering that these glasses contain a fairly high alkalis content, studies were conducted to examine the effect of alkali oxides on the strength characteristics of glass polymers. For this purpose, the dealcalization of finely ground glass waste was carried out by extracting alkalis in distilled water at a temperature of 70 °C, which was followed by drying at 100 °C.

Comparing the results presented in Table 2 and Table 4, one can note a tendency toward a decrease in the tensile strength of samples with dealcalized glass powder.

To determine the influence of the alkaline component on the properties of glass polymer, the method [53,58] was used to determine the alkali content on its surface. First, we determined the alkaline activity (AA) of glass powder before and after dealcalization and the level of alkaline activity of solid glass polymers of various compositions. Alkaline activity was determined on disk samples with a diameter of about 140 mm and a thickness of 3 mm. The exposure time of the extraction process was 5 s. Table 5 presents the research results.

First of all, it should be noted that the dealcalization of finely ground waste glass reduces its alkaline activity by 2.6 times in both sodium and potassium. When glass powder is introduced into the glass polymer at a 1:1 ratio, its alkaline activity decreases by 12.4% in sodium and by 50.8% in potassium compared to SWG-100. Similarly, when glass powder is introduced into the composition of a glass polymer with PWG-100 at the same ratio of water glass to glass powder, an almost twofold decrease in alkaline activity for both potassium and sodium is observed. Thus, by introducing dealcalized glass powder into the composition of the glass polymer, it is possible to reduce the alkaline activity of sodium and potassium by 30% and 35–55%, respectively. The above results demonstrate that by conducting controlled dealcalization, one can regulate the alkaline activity of glass polymers that use waste glass as an aggregate.

**Figure 3:**
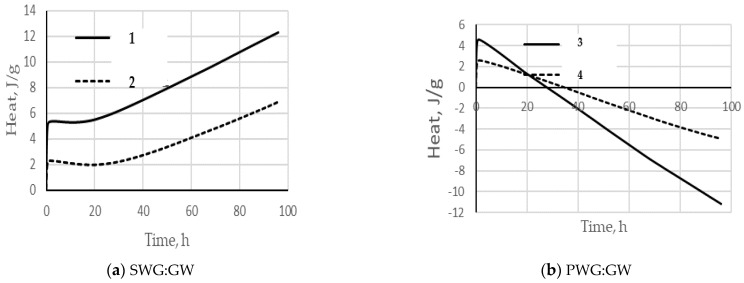
The process of heat release over time when adding waste glass (GW) to water glass (SWG or PWG) at different ratios: (**a**) 1—SWG:GW/1:1; 2—SWG:GW/1:2; (**b**) 3—PWG:GW/1:1; 4—PWG:GW/1:2.

The above data highlight the significant role of glass powder in enhancing the adhesion strength and cohesion of the glass polymer. It can be postulated that a chemical interaction occurs between the water glass and the finely ground fraction of glass waste, resulting in the formation of a solid-phase substance—a glass polymer with distinct structural and property differences from solidified water glass. To test this hypothesis, microcalorimetric studies were conducted using a microcalorimeter. Figure 3 illustrates the results of the heat release upon the contact of water glass with waste glass.

Analyzing the results presented in Figure 3, it should be noted that sodium and potassium water glasses react differently with finely ground glass. In the case of SWG, heat release is observed after 20 h after contact of SWG with GW for both the 1:1 and 1:2 ratios. In the second case, a decrease in the amount of heat by 2.7 times is observed. This may indicate that the exotherm of the process depends on the mass ratio of the reacting components with the main role played by the aggregate—finely ground waste glass.

As is known [37,59], finely ground glass waste belongs to the group of chemically stable glasses, the weak link of which is the alkaline component. In this case, alkaline cations modifiers are connected in the structural network by an ionic bond with a non-bridging oxygen anion. The value of this bond is about 600–1100 kJ/mol [60], which allows the alkaline cation, under certain conditions, to easily leave its place and take part in chemical reactions of glass with various chemical reagents present in the solution. Therefore, one would expect an increase in the alkaline activity of the glass polymer; however, as studies have shown, upon contact with glass powder with water glass, which is known to have an alkaline environment with a pH of 11–12, when glass powder is added, a decrease in alkaline activity is observed from 1.94 to 1.70 ppm/dm^2^. At the same time, the contribution of glass alkalis is almost 25 times lower than that of water glass; see Table 5. For all compositions given in Table 5, a decrease in the alkaline activity of glass polymers is observed with the introduction of finely ground glass. This contradiction can be explained by reducing the proportion of aqueous glass with a higher alkaline activity per unit surface when it is replaced with glass powder with a significantly lower alkaline activity. The same pattern is confirmed when the SWG:GW ratio increases from 1:1 to 1:2, as shown in Table 5.

As is known, alkalis belong to the reagents of the second group [56], which dissolve glass. In this case, the entire chemical component of the glass goes into solution. Thus, the chemical interaction of water glass with finely ground glass waste can be considered a process of slow dissolution of the latter in an alkaline glass polymer environment, which is accompanied by heat release, as shown in Figure 3.

In the case of potassium water glass, a completely different picture takes place; the interaction process is accompanied by heat absorption, as shown in Figure 3. This absorption may be associated with the replacement of potassium cations in water glass with sodium cations released by the glass powder with the formation of a phase of the Na_2_O composition, SiO_2_ nH_2_O. Thus, two phases may be present in an ionic solution of potassium water glass: ((Na_2_O^.^mSiO_2_) + (K_2_O^.^mSiO_2_))^.^nH_2_O. The combined presence of two types of alkalis in a water glass solution leads to a polyalkaline effect [61,62], which is expressed as a decrease in the alkaline activity of the mixture, Table 4.

The results presented in this article indicate that finely ground waste glass is an active aggregate in glass polymers based on water glass. The mechanism of interaction of the polymer with the alkaline component of the glass depends on the type of water glass. The result of this interaction is a change in the structure of the glass polymer, which is reflected in the strength cohesion, as shown in Table 3.

Since cohesive strength characterizes the mechanical strength of a material, the effect of glass powder on the cohesive strength of a glass polymer was evaluated by determining its mechanical strength. Table 6 presents the results of determining mechanical strength depending on the composition of the glass polymer and hardening time.

The results presented in Table 6 show that for composition N1, the increase in impact strength over 11 days is 5.2%; for N2, it is 5.4%; and for position 3, it is16.4%. The compressive strength increases by 2 times, 1.7 times and 2 times, respectively. Thus, the mechanical strength of the glass polymer increases as the curing time increases, which may be due to the compaction of the structure due to dehydration. In addition, it should be noted that the strength characteristics decrease with an increase in the amount of glass powder in the glass polymer composition.

It is known that any adhesive’s ability can be assessed by the adhesion value, i.e., the bond that occurs upon contact of the surface layers of two dissimilar bodies, in our case, liquid glass and glass powder substrate. As a substrate, finely ground glass waste is used on one side, and the surface of cement concrete is on the other side. Analysis of existing theories of adhesion suggests that in the case of water glass as an adhesive and fine glass as an aggregate, adhesion is due to their chemical interaction at the phase boundary. On the one hand, we have an ionic solution of aqueous glass, and on the other, we have solid glass particles, on the surface of which there are structural fragments of Si-O^−^Na^+^ with an ionic bond with a binding energy from 600 to 1100 kJ/mol and Si-OH with a hydrogen bond with a binding energy from 1 to 25 kJ/mol [60]. Comparing these values with the Si-O^−^ covalent bond energy (60–700 kJ/mol), we can conclude that the chemical bond between water glass and glass powder particles is provided to the greatest extent by the hydrated surface of the glass powder according to the following scheme:

Si-O-H(GW) + OH^−^(SWG) → Si-O-Si (phase boundary)+ H_2_O (1)

To a small extent, alkali metal cations will play a role in the formation of interfacial bonds according to the following scheme:

Si-O^−^ + Na(GW) + OH^−^(SWG) → Si-O-Si(phase boundary) + NaOH (2)

In the latter case, only cations with the smallest ionic bond values will take part in the formation of interfacial bonds.

In the case of the dealcalization of glass powder, the extraction of alkali cations from the surface of glass grains takes place, and their replacement is with hydrogen cations of water glass. Unfortunately, it is impossible to determine the degree of increase in the cohesive strength of the glass polymer, because tensile strength allows us to determine only the cohesive strength of concrete, which is significantly lower than the cohesive strength of the glass polymer.

When water glass or a glass polymer is applied to the surface of concrete, a mechanical interaction occurs in contrast to the contact of water glass with finely ground glass particles. The interaction is based on the penetration of water glass into the surface pores of the concrete and the formation of a so-called root layer, which ensures strong adhesion between the water glass and the concrete surface. In this case, the penetration depth depends on the pore size, the viscosity of the water glass, the surface tension value, etc. It can also be assumed that the adsorption factor of water glass influences the mechanical interaction between the internal walls of the pores and the surface of the concrete. Thus, in a glass polymer used as an adhesive, three adhesion mechanisms occur: mechanical, chemical, and adsorptive.

It should be noted that the structure of a glass polymer filled with waste glass is very similar to the structure of glass–ceramic materials, in which the solid crystalline phase makes up 80–90%, and the rest is a glassy layer. Moreover, the mechanical strength of such materials significantly exceeds the strength of the glassy layer; for example, the bending strength of glass is about 5–7 kg/mm^2^, and that of glass ceramic is 7–35 kg/mm^2^. A similar picture is observed in the case of glass polymer. The cohesive strength of sodium water glass as an interlayer is ~0.6 MPa, and the cohesive strength of glass polymer is >5 MPa, which exceeds the tensile strength of cement concrete.

## 4. Conclusions

This study has been successfully demonstrated by the potential of waste glass as an active aggregate in the production of glass polymers utilizing water glass as a binder. The research findings indicate the following.

The addition of finely ground waste glass to water glass significantly enhances the mechanical properties of the resultant glass polymer. This includes increases in both adhesive and cohesive strengths, which surpass those of traditional concrete, particularly after 7 days of curing where the tensile strength reaches up to 4.11 MPa.

The study identified optimal proportions of waste glass in adhesive grouts, balancing the chemical interactions and mechanical strengths. This optimization contributes to the efficiency of material use and sustainability in construction.

The modified glass polymers exhibit strong adhesion capabilities and are able to pull out fragments from the concrete substrate, suggesting significant potential for commercial applications in construction, especially in contexts requiring enhanced durability and strength.

The work established that the adhesion strength is greatly influenced by the alkaline activity of powdered glass waste, which, as is known, can fluctuate significantly due to poor-quality segregation. In this regard, to improve the quality of the glass polymer, it is proposed first to determine the alkaline activity of individual batches of waste glass and, if necessary, to adjust their alkaline activity by dealkalization from two components—sodium water glass and powdered silicate industrial waste glass.

By incorporating waste glass, the study contributes to waste reduction and promotes sustainability in the construction industry. The approach not only helps to manage glass waste but also reduces the environmental footprint of building materials.

## 5. Patents

PL237507B1 Method of determining the alkaline activity of cement products.

## Figures and Tables

**Table 1:** Characteristics of waste glass and sodium and potassium water glasses.

Materials	Oxides (wt %)						Siliceous Module	Density, g/cm^3^	Specific Surface, m^2^/g
	SiO_2_	Na_2_O	K_2_O	Al_2_O_3_	CaO + MgO	H_2_O			
Waste glass (GW)	72	10	4	1.0	12.0	-	-	2.43	3987
Sodium water glass (SWG)	26.38	8.02	-	-	-	65.6	3.4	1.45	-
Potassium water glass (PWG)	21.41	-	7.59	-	-	71.0	4.4	1.25	-

**Table 2:** The tensile strength of a glass polymer composite (GP) to concrete, depending on the composition.

N	Composition of Glass Polymer (GP) (wt %)			Tensile Strength f_t_, (MPa)				Zone Fracture	Note
	Sodium Water Glass (SWG)	Potassium Water Glass (PWG)	Waste Glass (GW)	7 Days	14 Days	21 Days	28 Days		
1	100	-	-	0.06	0.46	0.54	0.65	Fracture in GP	f_coh_ SWG
2	-	100	-	0.07	0.07	0.10	0.70	Fracture in GP	f_coh_ PWG
3	50.0	50.0	-	0.46	0.52	0.71	0.69	Fracture in GP	f_coh_ SWG + PWG
4	80.0	20.0	-	0.47	0.53	0.62	0.67	Fracture in GP	f_coh_ SWG + PWG
5	33.30	-	67.70	4.11	3.79	3.96	4.02	Fracture in concrete	f_coh.c_ *
6	-	33.30	67.70	3.66	3.13	3.21	3.61	Fracture in concrete	f_coh.c_ *

* f_coh.c_—cohesive strength of concrete.

**Table 3:** Tensile strength in bending of beams glued with various substances.

N	Adhesive Substance	f_t_, (MPa)
1	SWG-100%	1.4
2	PWG-100%	0.7
3	GP (SWG:GW/1:2)	6.5
4	GP (PWG:GW/1:2)	5.1
5	GP (SWG:GW */1:2)	2.3
6	GP (PWG:GW */1:2)	1.9

GP—glass polymer. * dealcalized glass powder after alkali extraction.

**Table 4:** The strength of glass polymer to concrete when using dealcalized glass powder.

N	Composition of Glass Polymer * (wt %)			Tensile Strength in Bending (MPa)				Zone Fracture	Note
	Sodium Water Glass (SWG)	Potassium Water Glass (PWG)	Waste Glass (GW)	7 Days	14 Days	21 Days	28 Days		
1	33.3	-	67.7	3.04	3.39	3.38	3.32	By concrete	f_coh_._c_
2	-	33.3	67.7	3.38	3.63	3.38	3.19	By concrete	f_coh.c_

* glass polymer composition (SWG:GW/1:2) and (PWG:GW/1:2).

**Table 5:** Alkaline activity of individual components and compositions of glass polymers of various compositions.

N	Composition of Solid Glass Polymer (wt %)	Alkaline Activity—AA (ppm/dm^2^)		∑Na^+^ + К^+^
		Na^+^	K^+^	
1	SWG-100	1.940	0.077	2.017
2	PWG-100	0.095	8.060	8.155
3	SWG:PWG/1:1	0.810	2.380	3.190
4	SWG:PWG/4:1	1.840	2.200	4.040
5	GW(powder)	0.080	0.022	0.102
6	GW-dA *(powder)	0.031	0.008	0.039
7	SWG:GW/1:1	1.700	0.190	1.890
8	SWG:GW/1:2	1.460	0.080	1.540
9	PWG:GW/1:1	0.380	3.970	4.350
10	PWG:GW/1:2	0.170	5.560	5.730
11	SWG:GWdA/1:1	1.350	0.055	1.405
12	PWG:GWdA/1:1	0.130	4.400	4.530
13	SWG:PWG:GW/1:1:2	1.460	2.290	3.750
14	SWG:PWG:GWdA/1:1:2	1.020	1.410	2.430

* dA—dealkalized waste glass (GW).

**Table 6:** Dependence of impact strength and compressive strength of glass polymer samples on composition and hardening time.

N	Composition of Glass Polymer	Impact Strength (J)			Compressive Strength (MPa)		
		by 5 Days	by 9 Days	by 16 Days	by 5 Days	by 9 Days	by 16 Days
1	SWG:GW/1:1	0.213	0.220	0.225	2.86	4.67	5.83
2	SWG:GW/1:2	0.222	0.230	0.234	2.86	4.70	5.85
3	SWG:GW/1:3	0.168	0.171	0.201	2.44	3.82	4.80

## Data Availability

The original contributions presented in the study are included in the article, further inquiries can be directed to the corresponding author.
